# Alleviation of drought stress in *Phyllostachys edulis* by N and P application

**DOI:** 10.1038/s41598-017-18609-y

**Published:** 2018-01-10

**Authors:** Zhi Zhuang Wu, Ye Qing Ying, Yuan Bin Zhang, Yu Fang Bi, An Ke Wang, Xu Hua Du

**Affiliations:** 1 Key Laboratory of High Efficient Processing of Bamboo of Zhejiang Province, China National Bamboo Research Center, State Forestry Administration, Hangzhou, 310012 Zhe Jiang China; 2 Nurturing Station for the State Key Laboratory of Subtropical Silviculture, Zhejiang A & F University, Lin’an, 311300 China; 3 Key Laboratory of Mountain Surface Processes and Ecological Regulation, Institute of Mountain Hazards and Environment, Chinese Academy of Sciences, Chengdu, 610041 China

## Abstract

The aim of this study was to explore whether nutrition supply can improve the drought tolerance of Moso bamboo under dry conditions. One-year-old seedlings were exposed to two soil water content levels [wellwatered, 70 ± 5% soil-relative-water-content (SRWC) and drought stress, 30 ± 5% SRWC] and four combinations of nitrogen (N) and phosphorus (P) supply (low-N, low-P, LNLP; low-N, high-P, LNHP; high-N, high-P, HNHP; and high-N, low-P, HNLP) for four months. Plant growth, photosynthesis, chlorophyll fluorescence, water use efficiency and cell membrane stability were determined. The results showed that drought stress significantly decreased total biomass, net-photosynthesis (Pn), stomatal-conductance (g_s_), leaf-chlorophyll-content (Chl_leaf_), PSII-quantum-yield (Φ_PSII_), maximum-quantum-yield-of-photosynthesis (Fv/Fm), photochemical-quenching-coefficient (qP), leaf-instantaneous-water-use efficiency (WUE_i_), relative-water-content (RWC), photosynthetic-N-use-efficiency (PNUE), and photosynthetic-P-use-efficiency (PPUE). N and P application was found to be effective in enhancing the concentration of leaf N, g_s_, and Pn while reducing the production of reactive oxygen species under both water regimes. Under LNHP, HNHP and HNLP treatments, the decreases in total biomass, Pn, Chl_leaf_ and Fv/Fm of drought-stressed were less evident than the decreases under LNLP. The study suggests that nutrient application has the potential to mitigate the drastic effects of water stress on Moso bamboo by improving photosynthetic rate, water-use efficiency, and increasing of membrane integrity.

## Introduction

Drought is one of the major abiotic stresses affecting plant growth worldwide^1,2^. Previous studies have found that drought stress decreased stomatal conductance (g_s_), leaf transpiration rate (E), and net photosynthesis (Pn)^3,4^. Drought also induced the generation of reactive oxygen species (ROS) and stimulated the activity of oxidative stress enzymes in leaf cells, e.g., increases in superoxidase^5,6^. Photosynthesis is the most crucial process for plant growth^7,8^. Many studies have used a decrease in the maximum quantum yield of photosynthesis (Fv/Fm) or a decrease in effective PSII quantum yield (Φ_PSII_) as indicators of the extent to which environmental stress has damaged the photosynthetic apparatus^9–12^. In addition, water use efficiency (WUE), the ability of the plant to produce dry matter/unit of water, is an important indicator of a plant’s resistance to drought stress. Drought stress generally increases WUE, which provides a fitness advantage in water-limited habitats^3,4,13–15^.

Nitrogen (N) and phosphorus (P) are primary macronutrients that play critical roles in various processes such as photosynthesis^16–18^ Nutrient addition plays a critical role in improving plant growth under drought stress because water deficit conditions constrain plants’ access to soil N^19^. Numerous studies have shown that fertilization may lessen the adverse effects of drought on plant growth by promoting the regulation of water use efficiency and enhancing the activities of antioxidant enzymes^20,21^. The interactive effects of water and N availability on photosynthesis have been extensively studied^22–25^. Previous studies found that nutrient combinations are more effective than the application of individual nutrients^26–31^. However, the interactive effects of drought and nutrition have not been thoroughly investigated in bamboo.

Moso bamboo (*Phyllostachys edulis*) is widely distributed in southern China^32^. The Moso bamboo forest in China has an area of 3.87 million ha, comprising 70% of the total bamboo forest area in China^33,34^. Moso bamboo is subjected to drought stress frequently, as irrigation water is not always available. Among the mineral nutrients used by plants nitrogen (N) demand is the highest^35^ since N can increase the photosynthetic rate^36,37^. There are many scholars concerned about the use of nitrogen fertilizer in Moso bamboo shoot production and its influence on Moso bamboo growth^38^, photosynthetic physiology^39^, soil and environmental effects^40^ of Moso bamboo forest. Phosphorus (P) also plays an important role in Moso bamboo growth^41^, for the deficiency of soil phosphorus in Moso bamboo forest is a common problem in southern China^42^. Also, the change of N and P ratio has important physiological and ecological significance for Moso bamboo forest management^43^. Inadequate fertilizer can potentially intensify the negative aspects of drought^44^. Nevertheless, the effects of the combination of N and P nutrients on the morphological, physiological, and biochemical characteristics of Moso bamboo plants are not well documented. In this study, we compared the growth, leaf photosynthesis, photosynthetic pigments, chlorophyll fluorescence, water use efficiency, activities of antioxidant enzymes, and lipid membrane peroxidation under four fertilization treatments and two water regimes in order to explore the responses of bamboo population to water and nutrients from the physiological ecology and try to find the optimal way for the improvement of drought tolerance in Moso bamboo. The objectives were as follows: (1) to monitor the growth and physiological changes in Moso bamboo under drought, N and P application and their combination and (2) to determine whether the N and P application can reduce the detrimental effects of drought stress.

## Results

### Biomass accumulation and photosynthesis

The accumulation of biomass decreased under drought stress. Moreover, the total biomass accumulation (DMA) exhibited a significant increase under LNHP, HNHP and HNLP treatments. In contrast, under LNHP, HNHP and HNLP with drought treatments, the decrease in total biomass accumulation was less evident than in the LNLP treatment (Table 1). Pn, g_s_ and E significantly decreased under drought treatments. The treatments of LNHP, HNHP and HNLP also resulted in smaller decreases of Pn, g_s_, and E under drought conditions (Table 1). Drought significantly increased the root to shoot ratio (RS) in LNLP plants, whereas the drought-induced increase in RS was less pronounced in HNHP, HNLP and LNHP plants (Table 1).

**Table 1:** Dry matter accumulation (DMA), root/shoot ratio (RS), net photosynthesis (Pn), stomatal conductance (g_s_) and transpiration (E) in the seedlings of Moso bamboo, as affected by drought, N, and P and their combination.

Watering	N level	P level	DMA (g)	RS (root shoot ratio)	Pn (μmol m^−2^ s^−1^)	g_s_ (mmol m^−2^ s^−1^)	E (mmol m^−2^ s^−1^)
30%	L	L	22.12 ± 2.00 f	2.42 ± 0.21a	1.65 ± 0.20 g	24.75 ± 1.93e	0.53 ± 0.05e
30%	L	H	22.12 ± 2.15 f	2.27 ± 0.12b	2.31 ± 0.18 f	33.27 ± 3.11 cd	0.70 ± 0.06d
30%	H	H	26.51 ± 2.08e	1.81 ± 0.09c	2.99 ± 0.12de	33.77 ± 3.91 cd	0.73 ± 0.07d
30%	H	L	24.04 ± 3.55fe	1.73 ± 0.04c	2.57 ± 0.08ef	30.62 ± 1.85 cd	0.67 ± 0.05d
70%	L	L	30.53 ± 2.05d	1.25 ± 0.08e	3.16 ± 0.23d	39.19 ± 3.98c	1.27 ± 0.11c
70%	L	H	34.83 ± 2.54c	1.24 ± 0.03e	3.91 ± 0.41c	58.93 ± 7.27b	1.51 ± 0.03b
70%	H	H	45.54 ± 1.40a	1.54 ± 0.09d	6.43 ± 0.36a	76.18 ± 11.41a	2.19 ± 0.02a
70%	H	L	39.84 ± 2.00b	1.43 ± 0.11d	4.56 ± 0.50b	84.93 ± 9.17a	1.46 ± 0.13b
		P:Fw	***	***	***	***	***
		P:Fn	**	***	***	*	***
		P:Fp	***	NS	***	***	***
		P:Fw × n	*	***	*	NS	***
		P:Fw × p	**	*	NS	***	***
		P:Fn × p	NS	NS	NS	**	**
		P:Fw × n × p	NS	**	**	*	***

Each value is the mean ± SE (n = 6–8). Values followed by the same letter in the same column are not significantly different at the P > 0.05 level according to Tukey’s test. Fw, watering effect; Fn, nitrogen effect; Fp, phosphorus effect; Fw × n, watering and nitrogen interaction effect; Fw × p, watering and phosphorus interaction effect; Fn × p, nitrogen and phosphorus interaction effect; Fw × n × p, watering, nitrogen and phosphorus interaction effect, as determined by analyses of variance. NS, not significant; *P < 0.05; **P < 0.01; ***P < 0.001.

### Chloroplast pigments and photochemical parameters

Drought decreased total chlorophyll content, Φ_PSII,_ Fv/FM, and qP, but increased NPQ. Significant interaction effects of drought × nutrient were found on the total chlorophyll content, Φ_PSII_, Fv/Fm, qP and NPQ. The LNHP, HNHP, and HNLP treatments resulted in significantly higher Φ_PSII_, Fv/Fm, and qP than the LNLP treatments (Table 2). The increases in NPQ in drought-stressed plants were less evident in the LNHP, HNHP, and HNLP treatments than in the LNLP treatment (Table 2).

**Table 2:** Chlorophyll fluorescence characteristics of Moso bamboo under different treatments.

Watering	N level	P level	Chlorophyll content (mg g^−1^ FW)	Φ_PSII_	Fv/Fm	qP	NPQ
30%	L	L	2.43 ± 0.17d	0.30 ± 0.03d	0.65 ± 0.04d	0.49 ± 0.05c	0.80 ± 0.03a
30%	L	H	2.31 ± 0.22d	0.37 ± 0.01c	0.62 ± 0.01d	0.53 ± 0.07c	1.13 ± 0.04b
30%	H	H	2.72 ± 0.06c	0.50 ± 0.02b	0.72 ± 0.03ab	0.67 ± 0.06b	0.94 ± 0.05c
30%	H	L	2.31 ± 0.16d	0.39 ± 0.04c	0.69 ± 0.03c	0.56 ± 0.05c	1.19 ± 0.03a
70%	L	L	3.15 ± 0.28b	0.56 ± 0.04a	0.77 ± 0.02a	0.76 ± 0.02a	0.41 ± 0.07e
70%	L	H	3.13 ± 0.06b	0.56 ± 0.02a	0.75 ± 0.02ab	0.74 ± 0.05a	0.74 ± 0.08d
70%	H	H	3.73 ± 0.13a	0.59 ± 0.05a	0.74 ± 0.02b	0.79 ± 0.08a	0.42 ± 0.05e
70%	H	L	3.20 ± 0.08b	0.57 ± 0.04a	0.76 ± 0.02a	0.75 ± 0.07a	0.62 ± 0.08d
		P:Fw	***	***	***	***	***
		P:Fn	*	***	**	***	**
		P:Fp	**	NS	NS	NS	NS
		P:Fw × n	**	NS	***	NS	NS
		P:Fw × p	NS	NS	*	NS	***
		P:Fn × p	NS	**	***	NS	NS
		P:Fw × n × p	***	***	NS	**	***

Each value is the mean ± SE (n = 6–8). Values followed by the same letter in the same column are not significantly different at the P > 0.05 level according to Tukey’s test. Fw, watering effect; Fn, nitrogen effect; Fp, phosphorus effect; Fw × n, watering and nitrogen interaction effect; Fw × p, watering and phosphorus interaction effect; Fn × p, nitrogen and phosphorus interaction effect; Fw × n × p, watering, nitrogen and phosphorus interaction effect, as determined by analyses of variance. NS, not significant; *P < 0.05; **P < 0.01; ***P < 0.001.

### Relative water content (RWC), WUE_i_, PNUE and PPUE

**Figure 1:**
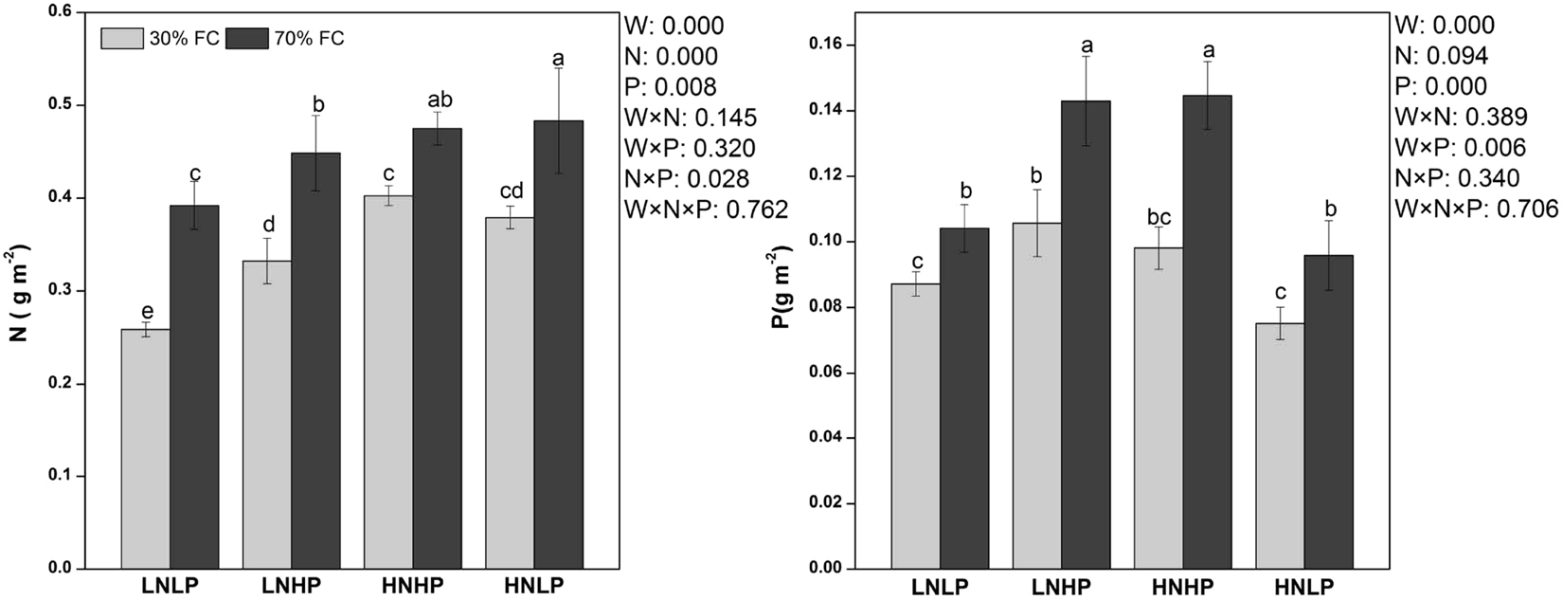
Leaf N content per leaf area (N) and leaf P content per leaf area of Moso bamboo under different treatments. Different letters above bars denote statistically significant differences between treatments at the P < 0.05 level according to Tukey’s test. The significance of the factorial analysis (analysis of variance): W, watering effect; N, nitrogen effect; P, phosphorus effect; W × N, watering and nitrogen interaction effect; W × P, watering and phosphorus interaction effect; N × P, nitrogen and phosphorus interaction effect; W × N × P, watering, nitrogen and phosphorus interaction effect. Watering regime [30% (grey bars) or 70% (black bars) of the field capacity] is shown.

Drought stress significantly reduced the RWC, whereas the drought-induced decrease in RWC was less evident in HNHP, HNLP, and LNHP treatments than in the LNLP treatment (Table 2). LNHP, HNHP, HNLP alleviated the negative effect of drought effects on RWC (Table 2). A significant interaction between drought and nutrition application was detected for WUE_i_ and PNUE, indicating that the drought-induced reduction in these parameters was greater under the LNLP treatment than under the HNHP, HNLP, and LNHP treatments (Table 3). Drought stress significantly decreased the leaf N and P content. The leaf N content exhibited a smaller reduction under LNHP, HNHP and HNLP than under LNLP (Fig. 1).

**Table 3:** Instantaneous water use efficiency (WUE_i_), photosynthetic N use efficiency (PNUE), photosynthetic P use efficiency (PPUE) and relative water content (RWC) of Moso bamboo under different treatments.

Watering	N level	P level	WUE_i_ (μmolmmol^−1^)	PNUE (μmol g^−1^s^−1^)	PPUE (μmol g^−1^s^−1^)	RWC (%)
30%	L	L	3.14 ± 0.42bc	6.39 ± 0.87c	18.99 ± 2.01e	66.31 ± 7.83c
30%	L	H	3.70 ± 0.29ab	7.74 ± 0.59c	24.42 ± 2.67de	75.89 ± 4.70b
30%	H	H	4.10 ± 0.36a	7.42 ± 0.19c	30.56 ± 2.22 cd	80.86 ± 1.50b
30%	H	L	3.85 ± 0.27ab	6.77 ± 0.14c	34.20 ± 1.70 cd	80.68 ± 0.78b
70%	L	L	2.88 ± 0.30bc	10.10 ± 0.89b	38.19 ± 4.02bc	90.82 ± 3.11a
70%	L	H	2.89 ± 0.34c	9.78 ± 1.08b	30.87 ± 4.18 cd	93.97 ± 2.44a
70%	H	H	2.93 ± 0.14c	13.53 ± 0.43a	44.60 ± 4.10ab	92.09 ± 1.43a
70%	H	L	3.13 ± 0.15c	9.44 ± 0.26b	48.32 ± 7.81a	91.79 ± 1.36a
		P:Fw	***	***	***	***
		P:Fn	NS	*	**	**
		P:Fp	NS	***	***	*
		P:Fw × n	NS	*	NS	**
		P:Fw × p	NS	NS	NS	NS
		P:Fn × p	NS	**	NS	*
		P:Fw × n × p	NS	**	NS	NS

Each value is the mean ± SE (n = 6–8). Values followed by the same letter in the same column are not significantly different at the P > 0.05 level according to Tukey’s test. Fw, watering effect; Fn, nitrogen effect; Fp, phosphorus effect; Fw × n, watering and nitrogen interaction effect; Fw × p, watering and phosphorus interaction effect; Fn × p, nitrogen and phosphorus interaction effect; Fw × n × p, watering, nitrogen and phosphorus interaction effect, as determined by analyses of variance. NS, no significant; *P < 0.05; **P < 0.01; ***P < 0.001.

### Enzyme activities and cellular damage

**Figure 2:**
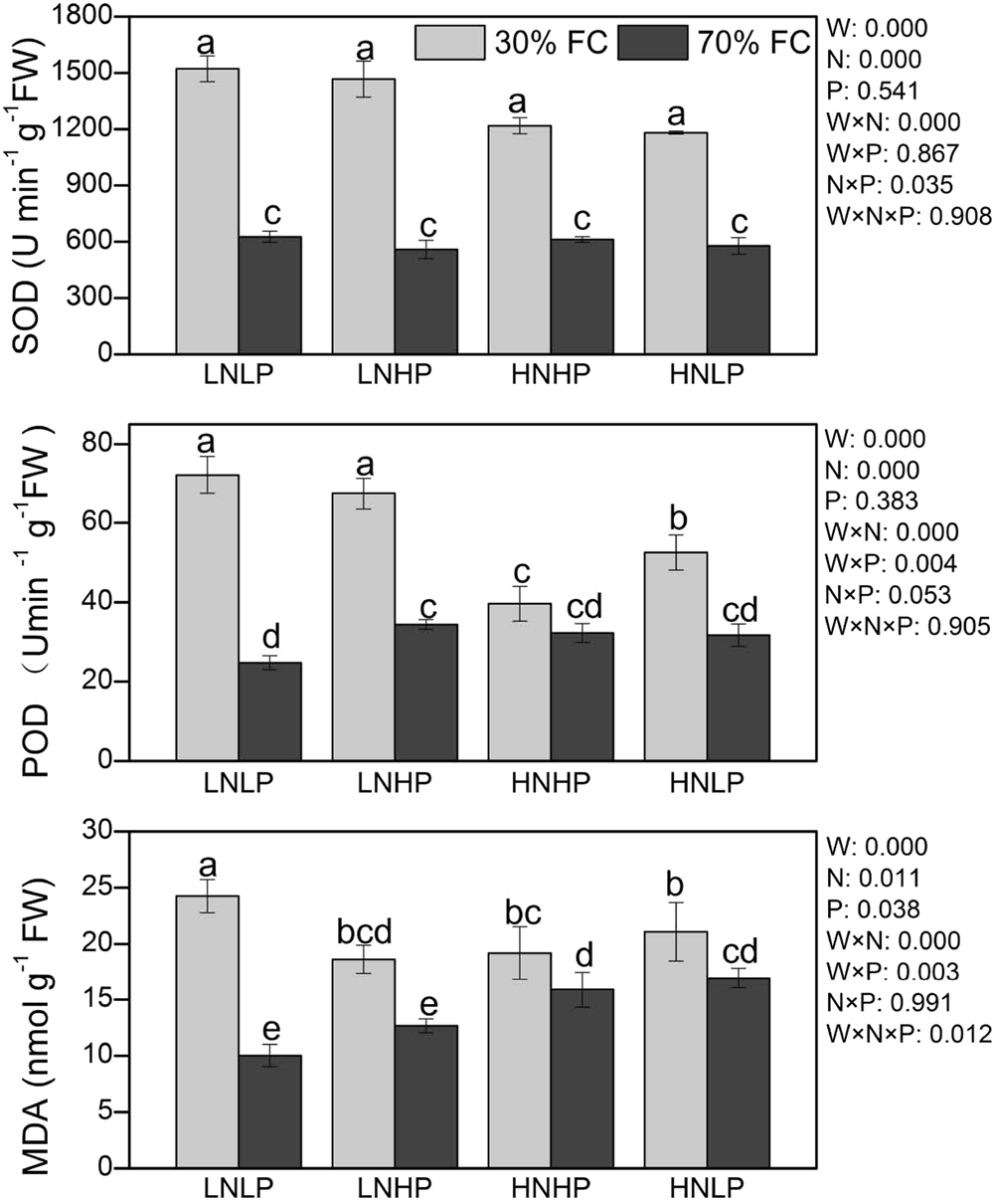
Superoxide dismutase (SOD), peroxidase activity (POD) and malondialdehyde (MDA) of Moso bamboo under different treatments. Different letters above bars denote statistically significant differences between treatments at the P < 0.05 level according to Tukey’s test. The significance of the factorial analysis (analysis of variance): W, watering effect; N, nitrogen effect; P, phosphorus effect; W × N, watering and nitrogen interaction effect; W × P, watering and phosphorus interaction effect; N × P, nitrogen and phosphorus interaction effect; W × N × P, watering, nitrogen and phosphorus interaction effect. Watering regime [30% (grey bars) or 70% (black bars) of the field capacity] is shown.

The oxidative stress resulting from drought was evident from the significant increases detected in MDA contents. Drought resulted in significantly higher POD and SOD activities and higher MDA. LNHP, HNHP, and HNLP plants had a significantly lower MDA content than LNLP plants. LNHP, HNHP and HNLP alleviated the damage resulting from drought stress on MDA. In addition, LNHP, HNHP and HNLP decreased the enhancement of SOD and POD under drought stress (Fig. 2).

## Discussion

Drought stress caused a pronounced inhibition of growth in Moso bamboo. In contrast, N and P application significantly promoted growth under the two watering regimes. On the one hand, the drought stress decreased the biomass, which might be due to the reduced photosynthates^2^. Drought significantly decreased gas exchange parameters, such as Pn, g_s_ and E, as reported in many previous studies^38,45,46^. Furthermore, the decreases in maximum quantum yield of photosynthesis (Fv/Fm) and effective photosystem II quantum yield (Φ_PSII_) indicated that structural injuries occurred in photosystem II primary photochemistry in Moso bamboo, as reported by Tezara *et al*. (2005) in two xerophytic shrubs. These studies have also shown that drought significantly decreased chlorophyll pigment contents, which coincides with the low photosynthesis rate under drought conditions. Therefore, it can be concluded that the reduction in photosynthesis rate under drought conditions may be due to stomatal limitation and PSII impairments. On the other hand, generally, drought can disrupt the electron transport chain, giving rise to oxidant stresses and inducing the production of ROS, which are harmful to organelles including the chloroplast, mitochondrion and peroxisome^47^. Specific enzymes, such as SOD and POD, protect cells from oxidative stress. In the present study, SOD and POD activities were increased by drought stress^1,43,48^. These findings suggest that drought alters enzymatic defence reactions to enhance the elimination system for ROS in Moso bamboo.

N and P may be involved in mitigating drought stress by improving dry matter production and its distribution between root and shoot. Previous studies on the interactive effects of N availability and stress conditions have produced various results, ranging from increased sensitivity to stress to decreased sensitivity to stress conditions^49^. Greater growth with nitrogen supply was generally observed when water was less limiting due to a higher photosynthetic rate or water use efficiency (WUE). No growth response to N supply under water stress was observed, however, due to the physiological or structural changes^50^. Such variation in results can be explained by the fact that the N effect on drought stresses depended mainly on the exposure duration, soil status, and nutrient requirements of species^49,51,52^. In the present study, our results indicate that the effects of the interaction between water and fertilization on plant dry mass accumulation were significant. In addition, the results showed that fertilization treatments increase the growth of roots less than they promote the growth of shoots, as the values of the root to shoot ratio decreased in conditions with fertilization compared to conditions without fertilization despite the same watering regimes.

N and P addition may help regulate photoassimilates and membrane integrity to minimize the effects of drought stress. On the one hand, RWC and the photosynthesis rate were more reduced while MDA less reduced in LNLP plants than in HNHP, HNLP, or LNHP plants under drought conditions, suggesting a positive role of N and P addition in minimizing the effects of drought stress by monitoring photoassimilates through increasing membrane integrity and decreasing photo-oxidation, as proposed by Akram *et al*. 2009. Furthermore, the improved stomatal conductance, which was also observed in HNHP, HNLP, and LNHP plants under drought-stressed conditions in this study, indicates that N and P application can reduce stomatal resistance and enhance CO_2_ assimilation^2^. N addition and P addition resulted in significantly higher Φ_PSII_, Fv/Fm, and qP, indicating that N and P application protects the photochemical efficiency from the damage caused by drought stress. In addition, the protection of photosynthesis and the tolerance of plants to drought stress can be stimulated by adopting strategies to increase the capacity for non-radiative dissipation of excitation energy as heat (NPQ)^53,54^. Moreover, an increase in transpiration due to N and P supply might be caused by the accumulation of solutes, absorption and utilization of water use, thereby increasing metabolic activities^55^. Our data showed that drought restrained the electron transport activity, with the restriction of PSII in HNHP, HNLP, and LNHP plants being less severe than in the LNLP plants, as reflected by the smaller decrease in qP. This result was consistent with the high CO_2_ assimilation detected under the same treatment. On the other hand, the combined N and P application significantly resulted in lower oxidative damage, measured with MDA, which ultimately increased the biomass of Moso bamboo under water deficit conditions. HNHP, LNHP, and HNLP treatments caused a significant decrease in the activity of SOD and POD enzymes under water deficit conditions, which was in contrast with the other findings^56^. Our results also highlight the lower pressure of oxidation and a reduced need for ROS removal (determined by low SOD and POD) in plants under HNHP, HNLP, LNHP treatments than in plants under LNLP treatments. The decreased activity of these enzymes suggested that Moso bamboo plants have the capacity to preserve the photosynthetic apparatus when grown under drought stress with N and P supplied. This fact is reinforced by the lower levels of lipid peroxidation and electrolyte leakage in the HNHP, HNLP, and LNHP treatments than in the LNLP treatment. Taken together, our findings reveal that the antioxidative system and cellular redox balance are more susceptible to disruption in LNLP than in HNHP plants.

Many studies have reported that water use efficiency increases with an increasing nutrient supply^57–60^, while other experiments have shown that N supply had no or negative effects on water use efficiency^61–63^. In our study, fertilization increased water use efficiency. This result is consistent with previous studies^58,59^. Our result suggests that high N alleviates the negative effects of drought stress on WUE by preventing cell membrane damage and through the overall enhancement of plant photosynthesis. It has been reported that drought stress significantly decreased plant growth by reducing the uptake of water and nutrients^64^. Similarly, our results show that drought stress significantly decreased leaf N and P concentrations, which might be attributed to the reduced N uptake by roots and its impaired transport from roots to shoots due to a restriction in transpiration rates^65^. Furthermore, the inhibitory effects of drought on PPUE were greater in LNLP than in HNHP, HNLP, and LNHP plants, which implies that N and P addition may promote plants to absorb and transport water and mineral nutrition under drought stress conditions. Nevertheless, N and P addition increased the total N and P concentration of leaves under both well-watered and water-stressed treatments. Similar results have been reported in other studies^66–68^.

In conclusion, drought stress reduced the total biomass, but N and P application, especially the combination of high N and high P, mitigated the adverse effects of drought. Under drought conditions, the reductions in total biomass were mainly caused by the reduction in photosynthesis rate, which may be due to stomatal limitation, which can alter enzymatic defence reactions to adapt to stress. Furthermore, N and P appear to be involved in mitigating drought stress by improving dry matter production driven by a higher photosynthetic rate, higher water use efficiency, and an increase in membrane integrity. Thus, the improved performance of Moso bamboo under nutrient treatments when exposed to drought may be mainly due to the maintenance of leaf water relations, oxidative damage alleviation and a greater capacity for the photoprotective process (indicated by increased RWC and decreased MDA). Therefore, in practice, the soil N and P status can be modified according to the background level of nutrients to improve drought tolerance. The combination of high N and high P may be an effective strategy to improve the drought tolerance of Moso bamboo seedlings. This study provides information useful for assessing seedling responses in nurseries, and certain ecophysiological responses of seedlings can be employed in modelling stand-scale performance. Such modelling is a key step needed to understand and improve the performance of Moso bamboo cultivation under different stresses in the field.

## Materials and Methods

### Plant materials and experimental design

One clump of one-year-old Moso bamboo (3–4 ramets) was planted in each of a total of 120 20-L plastic pots. The pots were filled with a mixture of loamy soil and perlite (3:1, v:v). The properties of the soil mixture used in the present study were as follows (based on kg^−1^ dry soil): pH 6.63, total N 783.65 mg, hydrolysable N 75.84 mg, total P 408.45 mg, available P 6.26 mg, total potassium 13.73 g, organic matter 23.24 g. There was one plastic tray for each pot to prevent nutrient loss. Seedlings were grown in a controlled environment room at Zhejiang A & F University (30°23′N, 119°72′E), China. The seedlings were grown in a greenhouse for four months in a semi-controlled environment. Treatments were administered from May 10 to September 10, 2015. During that period, the daytime temperature, night-time temperature and relative humidity ranged from 20–30 °C, 12–16 °C, and 45–80%, respectively. Before the experiment started, to keep the seedlings well watered, we irrigated the pots once every three days. Measurements of various morphological, physiological, and biochemical parameters were performed at the end of the experiment. The experiment was a completely randomized design with eight factorial combinations of two levels of water stress (well watered, water stressed) and four levels of nutrition (low N, low P; low N, high P; high N, low P; high N, high P). This experiment included 3 replications per treatment and 5 plants per replication. There were 2 levels of water treatments: normal water control (watered and maintained at 70 ± 5% field capacity) and drought stress (watered and maintained at 30 ± 5% field capacity). Water was added at 16:00–18:00 every 3 d according to the weight method. Under each water condition, there were also 4 nutrient combinations per type: (1) low N and P (N, 2.8 g/pot; P, 1.6 g/pot), LNLP; (2) low N and high P (N, 2.8 g/pot; P, 4.8 g/pot), LNHP; (3) high N and low P (N, 8.4 g/pot; P, 1.6 g/pot), HNLP; (4) high N and P (N, 8.4 g/pot; P, 4.8 g/pot), HNHP. These concentrations agree well with the conditions experienced by this species in the field. N and P were added weekly in the forms of NH_4_NO_3_ and NaH_2_PO_4_ solutions of 200 mL per pot at each application, resulting in the target application rate based on the container surface area. Every 2 weeks, all other nutrients were added in constant, non-limiting amounts.

### Growth measurement

At the end of the experiment, the plants were harvested and the shoots and roots were separated. The tissues were briefly rinsed with deionized water, oven dried at 70 °C for at least 48 h, weighed, and ground into fine powder. The dry weight (DWA) of shoots (leaves, stems) and roots was used to calculate the shoot/root ratio.

### Gas exchange and chlorophyll fluorescence measurements

The third or fourth fully expanded and exposed young leaves were selected for gas exchange and chlorophyll fluorescence measurements. We selected 5 samples per treatment. The net photosynthesis rate (Pn), E, and stomatal conductance (g_s_) were measured with a LI-COR 6400XT portable photosynthesis system (LI-COR Biosciences, Inc., Lincoln, USA) during a sunny day between 9:30 to 11:00. In this closed system, the ambient CO_2_, photosynthetic photo flux, relative humidity, and temperature were controlled at 350 µmol·mol^−1^, 1000 µmol·m^−2^·s^−1^, 60%, and 28 °C, respectively. The CO_2_ in the closed system was provided by a dedicated CO_2_ steel cylinder for the LI-COR 6400 instrument. The light was provided by an LED red-blue light chamber, which, unlike natural light, would supply a stable light source. The photosynthetic N use efficiency (PNUE) and photosynthetic P use efficiency (PPUE) were calculated as the ratio between photosynthetic rate and leaf N and P concentration, respectively. The instantaneous water use efficiency (WUE_i_) was determined by the ratio of net photosynthesis rate to transpiration.

Chlorophyll fluorescence was measured with a mini-PAM chlorophyll fluorometer (Walz, Effeltrich, Germany). We selected 10 samples for chlorophyll fluorescence measurement. The maximum quantum yield of photosynthesis (Fv/Fm), photochemical quenching coefficient (qP), effective PSII quantum yield (Φ_PSII_), and non-photochemical quenching (NPQ) were calculated as described by van Kooten and Snel (1990). After these treatments, the fresh leaves were cut immediately and extracted in 80% (v/v) chilled acetones and quantified using a spectrometer (Unicam UV-330, Unicam, Cambridge, UK) at wavelengths of 470 nm, 646 nm, and 663 nm for chlorophyll determination.

### Nitrogen (N) and phosphorus (P) analysis

The dried samples were ground to a fine powder and passed through a mesh (pore diameter ca. 275 µm). The N concentrations in these tissues were determined via flash combustion using a Carlo-Erba EA 1108 analyser. The leaf P concentration was determined through persulfate oxidation followed by colourimetric analysis (Schade *et al*. 2003).

### Enzyme extraction and assay

Fresh leaf samples on the third place of bamboo were collected for enzyme extraction. Enzymes were extracted at 4 °C from approximately 0.2-g leaf samples with 100 mM phosphate buffer (pH 7.8). This buffer contained 0.1 mM MEDTA, 1% (v/v) polyvinylpyrrolidone (PVP), 0.1 mM phenylmethylsulfonyl fluoride (PMSF), and 0.2% (v/v) Triton X-100. Extracting solutions were centrifuged at 6,000 × g for approximately 30 min. The supernatants were used for the measurements of superoxide dismutase (SOD) and peroxidase (POD).

SOD activity was assayed by the inhibition of the photochemical reduction of β-nitro blue tetrazolium chloride (NBT) (Dhindsa *et al*., 1980). One unit of SOD was defined as the amount of enzyme necessary to inhibit the reduction of cytochrome C by 50% at 560 nm. The reaction mixture had a total volume of 3.0 mL, containing 0.3 mL each of phosphate buffer (100 mM, pH 7.8), L-methionine (150 mM), 0.4 mL NBT (600 µM), riboflavin (20 µM), EDTA-Na^2^ (0.1 µM), and 1.5 mL extracting supernatants. The extracting supernatants were displaced by a phosphate buffer in control samples. The controls avoided the irradiance of light. The reaction was carried out for 20 min under irradiance of 4000 lx provided by a white fluorescent lamp. SOD was measured at 560 nm with a Shimadzu UV-2550 spectrophotometer (Kyoto, Japan).

POD activity was measured with guaiacol at 470 nm (Zhou, 2000). The reaction mixture contained potassium phosphate buffer (100 mM, pH 7.0), guaiacol (40 mM), H_2_O_2_ (10 mM), and enzyme extract. To calculate POD activity, we began recording changes in the mixture absorbance at 470 nm 30 s after the reaction had started and continued at 30-s intervals for a total of 3.0 min.

Malondialdehyde (MDA) content was determined with the thiobarbituric acid method (TBA) (Li, 2003). The reaction mixtures contained 2 mL supernatant and a 2-mL mixture of TBA (0.6%, v/v) and trichloroacetic acid (TCA, 10%, v/v). The mixtures were heated for 25 min at 100 °C. Then, mixtures were centrifugalized at 5000 × g for 20 min after they cooled. The supernatant was recorded at 532, 600 and 450 nm, respectively, with spectrophotometer. The MDA content was calculated using the following formula: C (μM) = 6.45 (OD_532_−OD_600_)−0.56OD_450_.

### Statistical analyses

Statistical analyses were conducted with the SPSS statistical software package version 11.5 for Windows. Three-way ANOVAs were applied to evaluate the effects of water, N, and P and the interaction among the three factors. Before ANOVAs, data were checked for normality and homogeneity of variances and log transformed to correct deviations from these assumptions when needed. Post hoc comparisons were tested using Tukey’s test at a significance level of P* < *0.05.

### Availability of materials and data

The datasets generated during and analyzed during the current study are available from the corresponding author on reasonable request.
